# Amorphous metal oxide semiconductor thin film, analog memristor, and autonomous local learning for neuromorphic systems

**DOI:** 10.1038/s41598-020-79806-w

**Published:** 2021-01-12

**Authors:** Mutsumi Kimura, Ryo Sumida, Ayata Kurasaki, Takahito Imai, Yuta Takishita, Yasuhiko Nakashima

**Affiliations:** 1 Graduate School of Science and Technology, Nara Institute of Science and Technology (NAIST), Takayama, Ikoma 630-0192 Japan; 2 Graduate School of Science and Technology, Ryukoku University, Seta, Otsu 520-2194 Japan; 3 Department of Electronics and Informatics, Ryukoku University, Seta, Otsu 520-2194 Japan

**Keywords:** Electrical and electronic engineering, Materials for devices, Computer science

## Abstract

Artificial intelligence is a promising concept in modern and future societies. Presently, software programs are used but with a bulky computer size and large power consumption. Conversely, hardware systems named neuromorphic systems are suggested, with a compact computer size and low power consumption. An important factor is the number of processing elements that can be integrated. In the present study, three decisive technologies are proposed: (1) amorphous metal oxide semiconductor thin films, one of which, Ga–Sn–O (GTO) thin film, is used. GTO thin film does not contain rare metals and can be deposited by a simple process at room temperature. Here, oxygen-poor and oxygen-rich layers are stacked. GTO memristors are formed at cross points in a crossbar array; (2) analog memristor, in which, continuous and infinite information can be memorized in a single device. Here, the electrical conductance gradually changes when a voltage is applied to the GTO memristor. This is the effect of the drift and diffusion of the oxygen vacancies (Vo); and (3) autonomous local learning, i.e., extra control circuits are not required since a single device autonomously modifies its own electrical characteristic. Finally, a neuromorphic system is assembled using the abovementioned three technologies. The function of the letter recognition is confirmed, which can be regarded as an associative memory, a typical artificial intelligence application.

## Introduction

Artificial intelligence is seen as a novel concepts in future societies with various applications in modern societies^1,2^. Neural networks are representative technologies that imitate the biological functions of the human brain^3–5^. Presently, software programs are used to practically realize artificial intelligence, in which conventional computers with high specifications are needed^6^. However, the computer size is unbelievably bulky, and the power consumption is incredibly large. Conversely, hardware systems are suggested as challenging solutions to mimic neural networks, which are named neuromorphic systems, in which customized materials, devices, circuits, algorithms, and systems are developed^7–15^. Hence, the computer size is expected to be excellently compact with significantly low power consumption. Based on brain science, one of the important factors for neuromorphic systems is the number of processing elements that can be integrated, but there have been few reports from this viewpoint.

In the present study, three decisive technologies are proposed for the astronomical large-scale integration in neuromorphic systems in the future, namely, (1) amorphous metal oxide semiconductor (AOS) thin films, one of which, Ga–Sn–O (GTO) thin film, is used. GTO thin film does not contain rare metals and can be deposited by a simple process, i.e., sputtering, at room temperature; (2) analog memristor, in which continuous and infinite information can be memorized in a single device; and (3) autonomous local learning, i.e., since a single device autonomously modifies its own electrical characteristic, extra control circuits are not necessary, which are sometimes larger than neuromorphic systems. In this paper, we explain these technologies thoroughly, and show an evaluation result when these are applied for associative memory as a typical artificial intelligence application.

## Results

### GTO memristor

**Figure 1:**
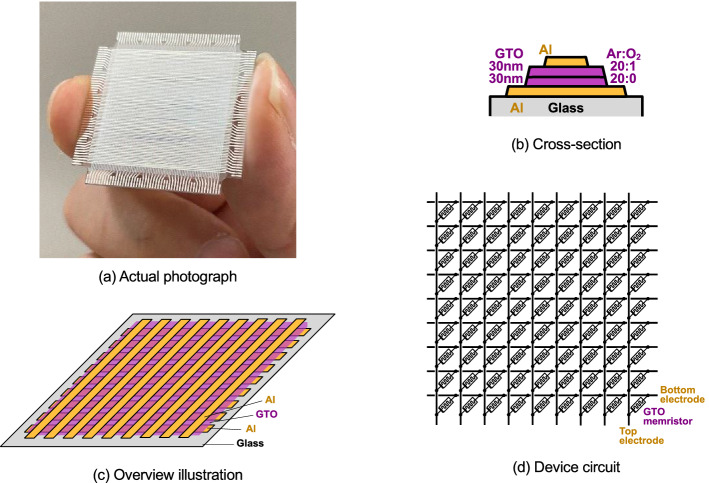
GTO memristor. (**a**) Actual photograph. (**b**) Cross-section. (**c**) Overview illustration. (**d**) Device circuit. A lower oxygen-poor and upper oxygen-rich GTO layers are stacked with different oxygen concentrations. The GTO memristors are formed at cross points in a crossbar array.

We create a GTO memristor using GTO thin films^16–18^. The GTO memristor is shown in Fig. 1. The actual photograph, cross-section, overview illustration, and device circuit are shown in Fig. 1a–d, respectively. Here, a lower oxygen-poor and upper oxygen-rich GTO layers are stacked with different oxygen concentrations. Because both the bottom and top electrodes are patterned to 80 bars orthogonal to each other, 80 × 80 = 6400 GTO memristors are formed at cross points in a crossbar array. Notably, the GTO thin film does not contain rare metals, and the GTO memristor is completed by a simple process at room temperature, which means the potential possibility for the astronomical large-scale integration in neuromorphic systems.

**Figure 2:**
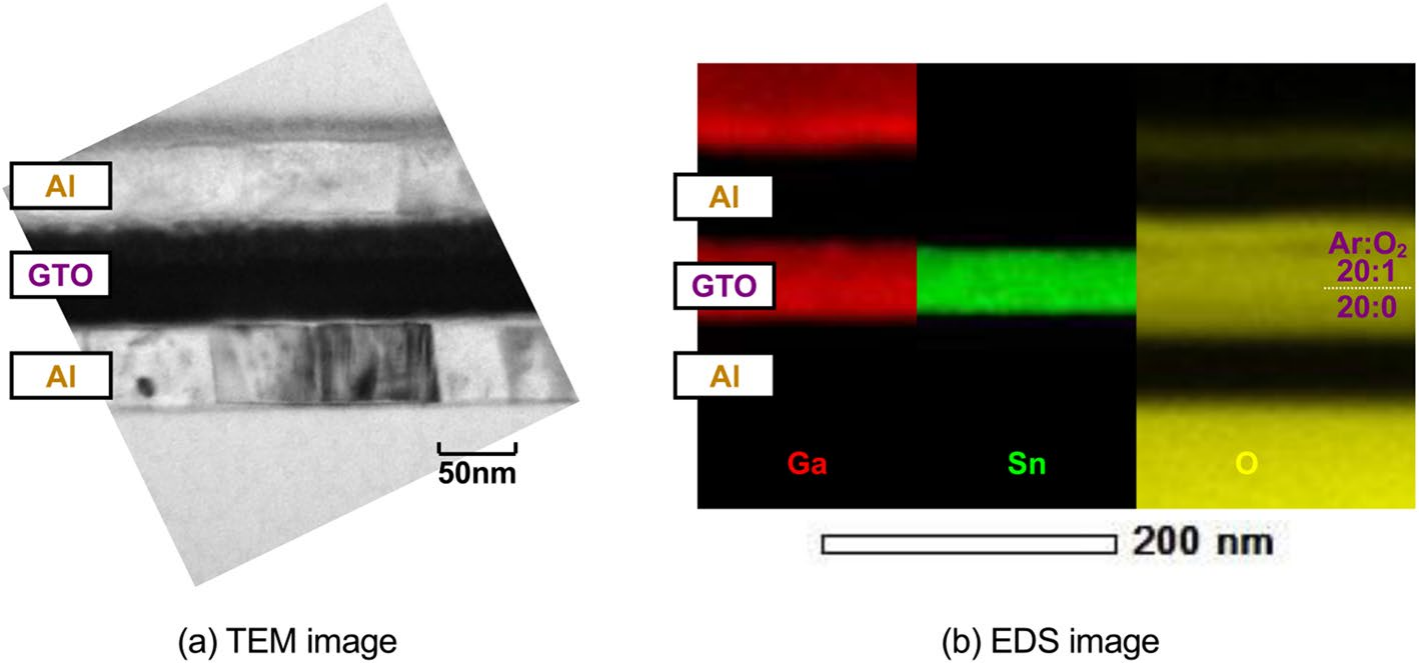
Cross-sectional image (**a**) TEM image. (**b**) EDS image. It is found that the cross section shown in Fig. 1b is certainly observed.

The cross-sectional image is shown in Fig. 2. The TEM and EDS images are shown in Fig. 2a, b, respectively. It is found that the cross section shown in Fig. 1b is certainly observed. The element analysis is shown in Table 1. Here, the element ratio is obtained from the EDS image. It is found that the oxygen concentration in the upper layer is higher than that in the lower layer. It has been also known that the oxygen concentration in the GTO thin film is surely dependent on the oxygen concentration in the sputtering gas^18^.

**Table 1:** Element analysis.

Layer	Ar:O_2_	Element ratio (%)			Ga:Sn:O
		Ga	Sn	O	
Upper	20:1	10.7	29.1	60.2	1.10:3:6.21
Lower	20:0	11.7	29.7	58.6	1.18:3:5.92

It is found that the oxygen concentration in the upper layer is higher than that in the lower layer.

**Figure 3:**
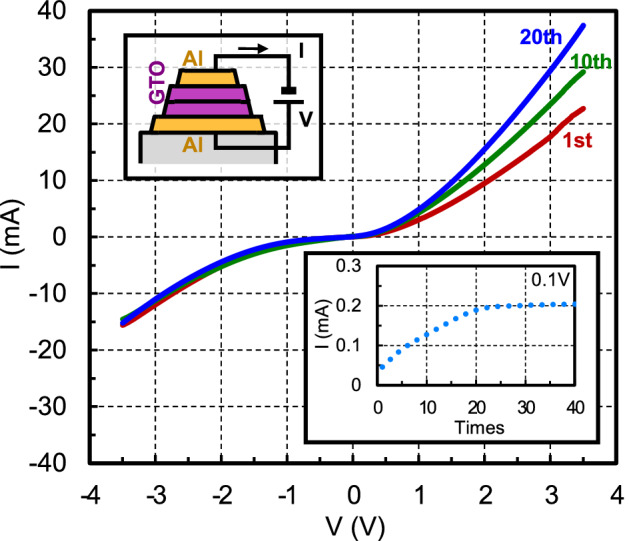
Analog memristor characteristic. The initial, 10th, and 20th measured electric currents are shown. It is found that the electrical conductance gradually increases when the voltage is positive.

The analog memristor characteristic is shown in Fig. 3. Here, the initial, 10th, and 20th measured electric currents are shown. Additionally, the relationship between the electric currents at a voltage of 0.1 V and the application times of the positive voltage is shown in the inset graph. It is found that the electrical conductance gradually increases when the voltage is positive until the conductance change is saturated after the application times of more than several tens, which means that the GTO memristor can be regarded as an analog memristor. Conversely, the electrical conductance is almost maintained when the voltage is negative, which means that the GTO memristor is itself non rewriteable.

**Figure 4:**
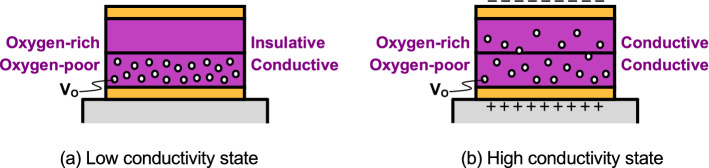
Working principle. (**a**) Low conductivity state. (**b**) High conductivity state. Initially, the upper oxygen-rich GTO layer includes few Vo and is insulative, which is the low conductivity state. When the voltage is applied, Vo drifts to the upper layer, and the upper layer becomes conductive, which is the high conductivity state.

The working principle is shown in Fig. 4. The low and high conductivity states are shown in Fig. 4a and (b), respectively. Initially, the lower oxygen-poor GTO layer includes several oxygen vacancies (Vo) and is conductive, whereas the upper oxygen-rich GTO layer includes few Vo and is insulative, and the electric current does not flow very much there, hence the low conductivity state. When the voltage is applied to the bottom electrode and positive, because Vo is positively charged, it is repulsed from the lower layer, drifts to the upper layer, and is diffused there. The upper layer becomes conductive, and the electric current flows very much, hence the high conductivity state. Because Vo gradually drifts, the electrical conductance gradually increases. Conversely, when the voltage is negative, because Vo is highly concentrated in the lower layer, it cannot be further concentrated there, which means that the electrical conductance is almost maintained. It should be noted that the small difference in the oxygen concentration causes the big difference in the electrical conductivity because the electrical characteristics are quite sensitive, and it is difficult to observe the difference in the oxygen concentration between the low and high conductivity states for the same reason. Notably, continuous and infinite information can be memorized in the GTO memristor, which means again the potential possibility for the astronomical large-scale integration in neuromorphic systems. Otherwise, a lot of digital binary memories must be prepared.

### Neuromorphic system

**Figure 5:**
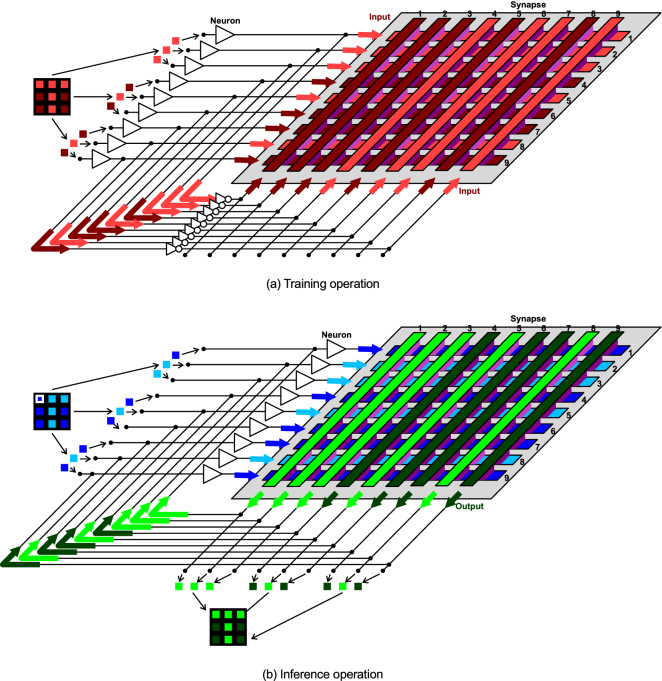
Neuromorphic operation. (a) Training operation. (**b**) Inference operation. During the training operation, the voltage that corresponds to the pattern to be memorized is applied to the bottom and top electrodes in the crossbar array. In (**a**), the bright purple at the cross points indicates that the electrical conductance increases, whereas the dark purple indicates that the electrical conductance is maintained. During the inference operation, the voltage that corresponds to the pattern slightly distorted from the memorized pattern is applied only to the bottom electrodes. Also in (**b**), the bright purple indicates that the electrical conductance increases, whereas the dark purple indicates that the electrical conductance is maintained. Consequently, some voltage is finally output after the transient behavior, which is a revised pattern.

Next, we assemble a neuromorphic system using the GTO memristors, which is a kind of full connection-type Hopfield neural network^19,20^. The neuromorphic operation is shown in Fig. 5. The training and inference operations are shown in Fig. 5a, b, respectively. During the training operation, the voltage that corresponds to the pattern to be memorized is applied to the bottom and top electrodes in the crossbar array. The electrical conductance changes depending on the voltage applied between the bottom and top electrodes. In Fig. 5a, the bright purple at the cross points indicates that the electrical conductance increases, whereas the dark purple indicates that the electrical conductance is maintained. During the inference operation, the voltage that corresponds to the pattern slightly distorted from the memorized pattern is applied only to the bottom electrodes. Also in Fig. 5b, the bright purple indicates that the electrical conductance increases, whereas the dark purple indicates that the electrical conductance is maintained. Consequently, some voltage is finally output after the transient behavior, which is a revised pattern. Notably, because the GTO memristor autonomously modifies its own electrical characteristic, extra control circuits are not necessary, which means that the training and inference operation can be regarded as autonomous local learning. It should be noted that the revised pattern is determined by the conductance balance between all the GTO memristors. Even if the electrical conductance of a GTO memristors increases, if that of another one increases further, the impact of the latter is greater. Therefore, the training operation can be continued until the conductance change is saturated. As a result, although the GTO memristor is itself non rewriteable as written above, the neuromorphic system can be rewritable.

**Figure 6:**
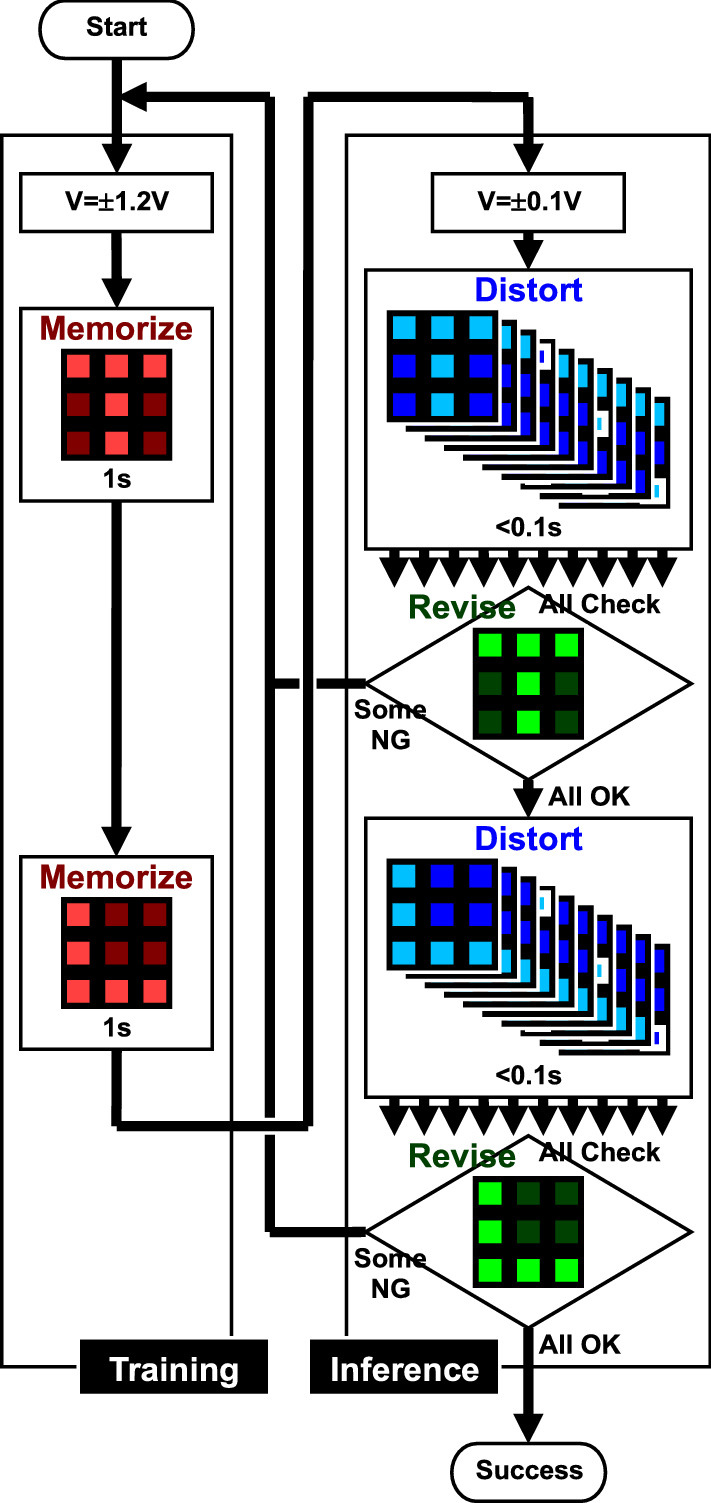
Evaluation flowchart. During the training operation, a relatively high voltage is applied. Two letter patterns are input for some time in sequence. During the inference operation, a relatively low voltage is applied. Slightly distorted patterns are input for a moment, and it is confirmed that the revised patterns are the same as the memorized pattern for all distorted patterns. If the revised pattern is different from the memorized pattern, the flowchart is repeated.

The evaluation flowchart is shown in Fig. 6. During the training operation, a relatively high voltage is applied so that the electrical conductance changes. Because a high voltage is applied, the device temperature may rise and accelerate the electrical conductance change in addition to the high voltage itself. Two letter patterns, “**T**” and “**L**,” are input for some time in sequence. During the inference operation, a relatively low voltage is applied so that the electrical conductance does not change. Multiple patterns slightly distorted from “**T**” and “**L**” are input for a moment, and it is confirmed that the revised patterns are the same as the memorized pattern for all distorted patterns. If the revised pattern is different from the memorized pattern, the flowchart is repeated until the revised patterns are the same as the memorized patterns, which can be regarded as letter recognition.

### Letter recognition

**Figure 7:**
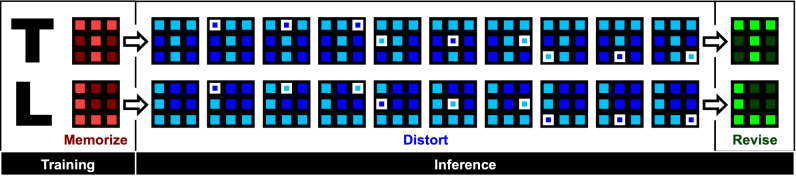
Letter recognition. It is found that the revised patterns are the same as the memorized patterns for all the distorted patterns.

The letter recognition is shown in Fig. 7. The flowchart of the training and inference operations is repeated a few tens times. It is found that the revised patterns are the same as the memorized patterns for all the distorted patterns. Notably, the letter recognition can be regarded as an associative memory, a typical artificial intelligence application.

## Conclusion

In conclusion, three decisive technologies were proposed for the astronomical large-scale integration in neuromorphic systems: (1) AOS thin films, one of which, GTO thin film, was used. GTO thin film does not contain rare metals and can be deposited by a simple process at room temperature. Here, oxygen-poor and oxygen-rich layers were stacked. GTO memristors were formed at cross points in a crossbar array; (2) analog memristor, in which continuous and infinite information can be memorized in a single device. Here, the electrical conductance gradually changed when a voltage was applied to the GTO memristor. This is the effect of the drift and diffusion of Vo; and (3) autonomous local learning, i.e., because a single device autonomously modifies its own electrical characteristic, extra control circuits are not required. Finally, a neuromorphic system was assembled using the abovementioned three technologies. The function of the letter recognition was confirmed, which can be regarded as an associative memory, a typical artificial intelligence application. These technologies may be necessary for the astronomical large-scale integration in neuromorphic systems in the future.

## Methods

### GTO memristor

The GTO memristor using the GTO thin films is created as follows. First, an Al thin film is deposited on a quartz glass substrate by vacuum evaporation as bottom electrodes. Next, a GTO thin film is deposited with a film thickness of 30 nm by radio frequency magnetron sputtering of a Ga:Sn = 1:3 GTO ceramic target in Ar gas at a deposition pressure of 1 Pa as a lower oxygen-poor GTO layer. Successively, in the same chamber without breaking the vacuum, another GTO thin film is deposited with a film thickness of 30 nm in Ar:O_2_ = 20:1 gas as an upper oxygen-rich GTO layer. Finally, another Al thin film is again deposited by vacuum evaporation as top electrodes. Because both the bottom and top electrodes are patterned to line of 150 μm and space of 150 μm, the memristor dimension is 150 × 150 μm. The TEM and EDS images are taken to observe the cross section and obtain the element ratio. The element ratio is obtained so that the Sn ratio is set to three, as shown at the rightmost row in Table 1, because Ga is implanted for the sample preparation and its concentration is not very accurate.

The analog memristor characteristic is measured as follows. A voltage is applied to the bottom electrode and scanned to either + 3.5 V or − 3.5 V multiple times, and the electric current is measured. The characteristic uniformity of general AOS devices has been also investigated^21–23^.

### Neuromorphic system

The neuromorphic operation is as follows. During the training operation, a two-dimensional letter pattern to be memorized is decomposed into a one-dimensional column pattern and input to neuron elements for a while. The voltage that corresponds to the one-dimensional column pattern is applied to the bottom electrodes in the crossbar array, and it is inverted and simultaneously applied to the top electrodes. During the inference operation, a two-dimensional pattern slightly distorted from the memorized pattern is input to neuron elements for a moment. The voltage that corresponds to the one-dimensional column pattern is applied only to the bottom electrodes. Consequently, some voltage is efficiently transmitted from the bottom electrodes to the top electrodes, and some voltage is generated at the top electrodes. The voltage is feedbacked to the bottom electrodes, and the voltage is finally output after the transient behavior. The one-dimensional column pattern that corresponds to the voltage is composed into a two-dimensional pattern, which is a revised pattern.

The evaluation flowchart is as follows. During the training operation, a voltage of ± 1.2 V is applied. Two letter patterns, “**T** and “**L**,” are input for 1 s in sequence. During the inference operation, a voltage of  ± 0.1 V is applied. First, multiple patterns slightly distorted from “**T** are input for a moment, some patterns are output, and it is confirmed that the revised patterns are the same as the memorized pattern for all distorted patterns. Next, multiple patterns slightly distorted from “**L**” are input, and it is again confirmed that the revised patterns are the same as the memorized pattern. If at least one revised pattern is different from the memorized pattern, the flowchart of the training and inference operations is repeated until the revised patterns are the same as the memorized patterns for all the distorted patterns. Here, mechanical relays are used to switch between ± 1.2 and ± 0.1 V, which is equivalent to the schematics in Fig. 5, but it is possible to implement such integrated circuits.
